# GPCA *vs.* PCA in Recognition and 3-D Localization of Ultrasound Reflectors

**DOI:** 10.3390/s100504825

**Published:** 2010-05-11

**Authors:** Carlos A. Luna, José A. Jiménez, Daniel Pizarro, Cristina Losada, José M. Rodriguez

**Affiliations:** Electronics Department, High Polytechnic School, Alcalá University, Alcalá de Henares, Madrid, Spain; E-Mails: jimenez@depeca.uah.es (J.A.J.); pizarro@depeca.uah.es (D.P.); cristina@depeca.uah.es (C.L.); jmra@depeca.uah.es (J.M.R.)

**Keywords:** principal component analysis (PCA), generalized principal component analysis (GPCA), reflector classification, times-of-flight (TOFs), ultrasonic sensors

## Abstract

In this paper, a new method of classification and localization of reflectors, using the time-of-flight (TOF) data obtained from ultrasonic transducers, is presented. The method of classification and localization is based on Generalized Principal Component Analysis (GPCA) applied to the TOF values obtained from a sensor that contains four ultrasound emitters and 16 receivers. Since PCA works with vectorized representations of TOF, it does not take into account the spatial locality of receivers. The GPCA works with two-dimensional representations of TOF, taking into account information on the spatial position of the receivers. This report includes a detailed description of the method of classification and localization and the results of achieved tests with three types of reflectors in 3-D environments: planes, edges, and corners. The results in terms of processing time, classification and localization were very satisfactory for the reflectors located in the range of 50–350 cm.

## 1. Introduction

The classification and localization of reflectors constitutes a fundamental task in the field of mobile robotics, since this information contributes in a decisive way to other higher level tasks, such as the generation of environment maps and the robot’s localization. With respect to the process of reflector classification, the techniques more broadly used are based on geometric considerations obtained from the TOFs for every reflector type [1,2]. An important inconvenience of the systems based on geometric considerations is their high dependence on the precision with which the measurements of the TOFs are carried out, and consequently, the classification results are strongly influenced by noise.

**Figure 1.**
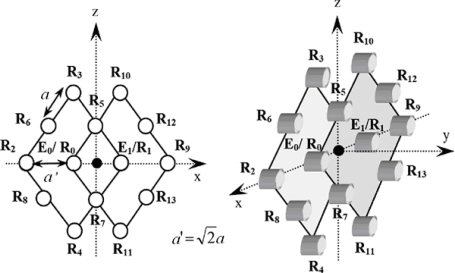
View of sensor structure proposed in [7].

Principal Component Analysis (PCA) has been used to reduce the dimension of data sets and object recognition in different works related to image processing [3–6]. The classification and localization technique for 3-D reflectors based on PCA using an ultrasonic sensor is explicitly discussed in [7], in which it is applied to 18 TOF values originated from a sensor that contains two emitter/receiver transducers and 12 receivers, (see Figure 1). The pulses emitted by E_0_/R_0_ are processed by itself (E_0_/R_0_) and by transducers E_1_/R_1,_ R_2_, R_3,_ R_4,_ R_5,_ R_6,_ R_7,_ and R_8_. The pulses emitted by E_1_/R_1_ are processed by transducers E_1_/R_1_, E_0_/R_0,_ R_5,_ R_7,_ R_9,_ R_10,_ R_11,_ R_12_, and R_13_. In [7–11] to reduce the number of transducers, the simultaneous emission of complementary sequences by two or more emitters is proposed.

The PCA is applicable only for data in vectorized representation. Therefore, the data obtained from a matrix sensor must have been previously converted to a vector form. A typical way to do this is the so-called “matrix to vector alignment”, which consists of concatenating all the rows in the matrix together to get a single vector. Figure 1 shows two matrix sensors of nine transducers. The TOFs *t*_0_*___*_2_, *t*_0_6_, *t*_0_*___*_3_, *t*_0_*___*_8_, *t*_0_*___*_0_, *t*_0_*___*_5_, *t*_0_*___*_4_, *t*_0_*___*_7_, *t*_0_*___*_1_ obtained from reception of sequence emitted by E_0_/R_0_ and the TOFs *t*_1_*___*_0_, *t*_1_*___*_5_, *t*_1_*___*_7_, *t*_1_*___*_9_, *t*_1_*___*_10_, *t*_1_*___*_11_, *t*_1_*___*_12_, *t*_1_*___*_13_ obtained from reception of sequence emitted by E_1_/R_1_ are aligned to get a single vector (1). Receiver R_2_ and R_8_ are neighbours in the sensor, while they are far away from each other in the vectorized representation. The same observations hold for positions R_0_ and R_6_, *etc.* Due to the vector alignment the spatial information is missed. Also it can be remarked that the original 3 × 3 matrixes are converted to an 18 × 18 scatter matrix in PCA, which leads to higher time and memory space costs:

(1) $$\begin{array}{l} \tau =[\underset{\mathrm{From}\;\mathrm{the}\;\mathrm{emitter}\;E_{0}}{\underset{︸}{\begin{array}{ccccccccc} t_{0\_2} & t_{0\_6} & t_{0\_3} & t_{0\_8} & t_{0\_0} & t_{0\_5} & t_{0\_4} & t_{0\_7} & t_{0\_1} \end{array}}}\\ \;{\underset{\mathrm{From}\;\mathrm{the}\;\mathrm{emitter}\;E_{1}}{\underset{︸}{\begin{array}{ccccccccc} t_{1\_0} & t_{1\_5} & t_{1\_10} & t_{1\_7} & t_{1\_1} & t_{1\_12} & t_{1\_11} & t_{1\_13} & t_{1\_9} \end{array}}}]}^{T} \end{array}$$

In [8], the sensorial structure is formed by four transducers which can simultaneously obtain 16 TOF values at every scanning process. These 16 TOF values are aligned in a vector that is used in the classification algorithm based on PCA.

In [12] the generalized PCA (GPCA) algorithm is used for image compression. The GPCA algorithm deals with the data in its original matrix representation and considers the projection onto a space, which is the tensor product of two vector spaces. In this paper, we use the GPCA for recognition and localization of ultrasound reflectors which aim to overcome the drawbacks in the traditional PCA. We used a matrix of 16 transducers (using four of the centre sensors as emitters/receivers and the other 12 as receivers). Thus, we can obtain up to four 4 × 4 matrices, each of which performs a classification procedure independently. The results of the four classification processes merge to give the final result.

The rest of this paper is organized as follows. Section 2 illustrates the proposed sensor structure. Section 3 describes the GPCA algorithm. Experimental results are presented in Section 4. And, conclusions are offered in Section 5.

## 2. Sensor Structure

**Figure 2.**
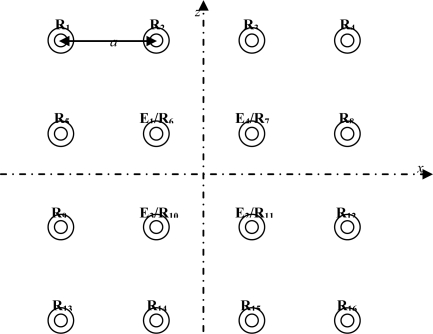
Sensor structure proposed in this paper.

The sensor structure used to extract information in a 3-D environment and to subsequently classify and locate ultrasonic reflectors with the obtained data (TOF sets) from echoes is shown in Figure 2.

The physical structure of the ultrasonic sensor consists of 16 transducers, four of them working as emitters and receivers (E_1_/R_6_, E_4_/R_7_, E_3_/R_10_ and E_2_/R_11_) and the others only working as receivers. All the transducers are located in a plane (plane *xz*), with the axial axis in the *y*-direction. Furthermore, the separation between transducers is determined by the distance *a* (*a* = 0.17 m).

With the proposed sensor structure it is possible to obtain up to 64 TOFs at every emission/scanning process. To do this, it is necessary to assign a different macrosequence to every transducer to encode their emissions. These macrosequences, obtained from complementary sets of sequences, allow simultaneous emission and reception to be carried out with all the transducers for the same scanned environment [8,11].

## 3. GPCA Algorithm

In this paper, the usage of GPCA is proposed to carry out the reflector classification using the measurements of 16, 32 and 64 TOFs provided by the ultrasonic sensors. This method maintains the spatial distribution of sensor data, which is represented by the TOFs in a matrix format (2):

(2) $$\tau_{e}=\left[\begin{array}{cccc} t_{1} & t_{2} & t_{3} & t_{4}\\ t_{5} & t_{6} & t_{7} & t_{8}\\ t_{9} & t_{10} & t_{11} & t_{12}\\ t_{13} & t_{14} & t_{15} & t_{16} \end{array}\right]$$

In (2), *t*_1_*… t*_16_ are the TOFs associated to each receiver and **τ***_e_* ∈ **IR***^r^* ^×^ *^c^* is the TOF matrix obtained for each emitter (*r* and *c* are the number of rows and columns of the sensor, respectively). Therefore, in our case we can obtain up to four matrices ( $\tau_{e}^{E1}$, $\tau_{e}^{E2}$, $\tau_{e}^{E3}$ and $\tau_{e}^{E4}$ for the emitters *E*1, *E*2, *E*3 and *E*4).

In GPCA we compute an optimal (*l*_1_, *l*_2_)-dimensional space, such that the projections of the data points (subtracted by the mean) onto this axis system have the maximum variance among all possible (*l*_1_, *l*_2_)-dimensional axes systems. Unlike PCA, the projections of the data points onto the (*l*_1_, *l*_2_)-dimensional axis system in GPCA are matrices, instead of vectors.

Let consider *S* = {***τ***_0_, ***τ***_1_, ***τ***_2_, …***τ***_n−1_} be a training set of *n* samples of TOF matrix. The mean TOF matrix of the set is defined by:

(3) $$\bar{\tau }=\frac{1}{n}\sum_{i=0}^{n-1}\tau_{i}$$

Matrices with mean zero are represented as:

(4) $$\Phi_{i}=\tau_{i}-\bar{\tau }$$

Then the variance of the projections of **Φ***_i_* onto the (*l*_1_, *l*_2_) dimensional axis system is defined as:

(5) $$\text{var}(L,R)=\frac{1}{n}{\sum_{i=0}^{n-1}\left\|L^{T}\Phi_{i}R\right\|}_{F}^{2}$$

where || ||*_F_* is the Frobenius norm and **L** ∈ IR^*r×l*_1_^ and R ∈ IR^*c*×*l*_2_^ are two matrices with orthonormal columns, such that the variance var(**L**,**R**) is maximum. The maximum value of (5) cannot be found in closed form and thus an iterative approach is needed:

♦ For a given **R**, the optimal matrix **L** consists of the *l*_1_ eigenvectors of the matrix **M_L_** which correspond to the largest *l*_1_ eigenvalues, where:
  (6) $$M_{L}=\sum_{i=0}^{n-1}\Phi_{i}{\mathrm{RR}}^{T}\Phi_{i}{}^{T}$$
♦ In the same way, given **L**, the matrix **R** consists of the *l*_2_ eigenvectors of the matrix **M_R_**, corresponding to the largest *l*_2_ eigenvalues, where:
  (7) $$M_{R}=\sum_{i=0}^{n-1}\Phi_{i}{}^{T}{LL}^{T}\Phi_{i}$$

In the realized experiments we used *l_1_* *= l_2_* = 2 (**L** ∈ IR^4 × 2^ and **R** ∈ IR^4 × 2^) and *l*_1_ *= l*_2_ = 1 (**L** ∈ **IR^4×1^** and **R** ∈ **IR^4×1^**).

To calculate the **L** and **R** matrices that maximize Equation 5, it is necessary to initially fix one of them. Fixing **L**, we can calculate **R** by computing the eigenvectors of the matrix **M_R_**, and then, with the calculated **R** we can then update **L** by computing the eigenvectors of the matrix **M_L_**. It is necessary to repeat the procedure until the result converges. The solution depends on the initial choice of **L** (**L**_0_). As it is recommended in [12], we use **L**_0_ = (**I**, 0)*^T^*, where **I** is the identity matrix. To measure the convergence of the GPCA procedure we use the root mean square error (*ζ*), defined as follows:

(8) $$\xi =\sqrt{\frac{1}{n}\sum_{i=0}^{n-1}{\left\|\Phi_{i}-{LL}^{T}\Phi_{i}{RR}^{T}\right\|}_{F}^{2}}$$

In the realized experiments for *ζ* = 10^−8^ the procedure converges within five iterations.

Once the transformation matrices **L** and **R** are determined and given a new TOF matrix **τ***_i_* to be classified, its zero-mean version **Φ***_i_* is transformed into the feature space as:

(9) $$\Omega_{i}=L^{T}\Phi_{i}R$$

Then we can reconstruct **Φ***_i_* as:

(10) $${\hat{\Phi }}_{i}\approx L\Omega_{i}R^{T}$$

The reconstruction error (*ε_R_*) for **Φ̂***_i_* can be computed as:

(11) εR=‖Φi−Φ^i‖F=‖Φi−LLTΦiRRT‖F

### 3.1. Offline Generation of the Classes

The objective of this work is to classify one of the three reflector types (plane, corner, and edge) and its approximate direction (azimuth angle *γ*, elevation angle *θ*) and distance (*r*) with respect to the frontal space of the sensor. Before beginning the classification process, it is necessary to create different classes, depending on the reflector type and its spatial location. Every class has two transformation matrices **L**, **R** associated to it. These matrices are referred as **L***^P^* and **R***^P^* for the plane, **L***^C^* and **R***^C^* for the corner, and **L***^E^* and **R***^E^* for the edge.

As it is stated in [7,8], we assume that the frontal space of the sensorial structure is formed by *Q* directions defined by (*γ_q_, θ_q_*), with *q* ∈ {1, 2,*…*, *Q*}. Along every direction *q*, there are *D* discrete distances referred to as *r_d_*, with *d* ∈ {1, 2,..., *D*}. To generate the transformation matrices associated to every direction class and every reflector type, the reflectors *{P*,*C*,*E}* have been located at every direction *q* and for all the *d* distances, obtaining the TOF vectors. In GPCA, to generate the transformation matrices associated with every direction and every reflector type ( $L_{q}^{P}$, $R_{q}^{P}$, $L_{q}^{C}$, $R_{q}^{C}$, $L_{q}^{E}$, $R_{q}^{E}$), the reflectors {*P*, *C*, *E*} have been located at every direction *q* and for all the *D* distances.

When more than one transducer is emitting, we use a matrix of TOF for each emitter, *i.e.*: $\tau_{e}^{E1}$, $\tau_{e}^{E2}$, $\tau_{e}^{E3}$ and $\tau_{e}^{E4}$ for the emitters *E*1, *E*2, *E*3 and *E*4. Therefore, it is necessary to compute the transformation matrices associated to every direction and every reflector type ( $L_{q}^{P}$, $R_{q}^{P}$, $L_{q}^{C}$, $R_{q}^{C}$, $L_{q}^{E}$, $R_{q}^{E}$) for each emitter.

### 3.2. Classification and Position Estimation of the Reflector

The strategy proposed in this paper to carry out the online classification process is to first classify the type and approximate direction in which the reflector is located and then to estimate its distance with respect to the frontal space of the sensor.

To classify the type and approximate direction of the reflector, we calculate the square reconstruction error (*ε*), using the transformation matrices associated to every direction *q* and every reflector type:

(10) $$\begin{array}{l} \left(\varepsilon_{q}^{P}\right)={\left\|\Phi_{q}^{P}-L_{q}^{P}L_{q}^{P^{T}}\Phi_{q}^{P}R_{q}^{P}R_{q}^{P^{T}}\right\|}_{F}^{2}={\left\|\Phi_{q}^{P}\right\|}_{F}^{2}-{\left\|L_{q}^{P^{T}}\Phi_{q}^{P}R_{q}^{P}\right\|}_{F}^{2}\\ \left(\varepsilon_{q}^{E}\right)={\left\|\Phi_{q}^{E}-L_{q}^{E}L_{q}^{E^{T}}\Phi_{q}^{E}R_{q}^{E}R_{q}^{E^{T}}\right\|}_{F}^{2}={\left\|\Phi_{q}^{E}\right\|}_{F}^{2}-{\left\|L_{q}^{E^{T}}\Phi_{q}^{E}R_{q}^{E}\right\|}_{F}^{2}\\ \left(\varepsilon_{q}^{C}\right)={\left\|\Phi_{q}^{C}-L_{q}^{C}L_{q}^{C^{T}}\Phi_{q}^{C}R_{q}^{C}R_{q}^{C^{T}}\right\|}_{F}^{2}={\left\|\Phi_{q}^{C}\right\|}_{F}^{2}-{\left\|L_{q}^{C^{T}}\Phi_{q}^{C}R_{q}^{C}\right\|}_{F}^{2} \end{array}$$

The reflector is classified as a plane if $\varepsilon_{q\;\text{min}}^{C}>\varepsilon_{q\;\text{min}}^{P}<\varepsilon_{q\;\text{min}}^{E}$, it is classified as an edge if $\varepsilon_{q\;\text{min}}^{C}>\varepsilon_{q\;\text{min}}^{E}<\varepsilon_{q\;\text{min}}^{P}$ and it is classified as a corner if $\varepsilon_{q\;\text{min}}^{P}>\varepsilon_{q\;\text{min}}^{C}<\varepsilon_{q\;\text{min}}^{E}$. The value of *q*, which corresponds to the minimum value of *ε_q_* determines the approximate direction of the reflector. When more than one emitter is being used, we determine the minimum value of reconstruction error in each direction for each emitter, and added to all these minimum values. The minimum values are taken with the same angle for all transmitter, otherwise the results will not classify correctly. For four emitters that is:

(11) $$\begin{array}{c} \varepsilon_{q\text{min}}^{P}={\left(\varepsilon_{q\text{min}}^{P}\right)}^{E1}+{\left(\varepsilon_{q\text{min}}^{P}\right)}^{E2}+{\left(\varepsilon_{q\text{min}}^{P}\right)}^{E3}+{\left(\varepsilon_{q\text{min}}^{P}\right)}^{E4}\\ \varepsilon_{q\text{min}}^{E}={\left(\varepsilon_{q\text{min}}^{E}\right)}^{E1}+{\left(\varepsilon_{q\text{min}}^{E}\right)}^{E2}+{\left(\varepsilon_{q\text{min}}^{E}\right)}^{E3}+{\left(\varepsilon_{q\text{min}}^{E}\right)}^{E4}\\ \varepsilon_{q\text{min}}^{C}={\left(\varepsilon_{q\text{min}}^{C}\right)}^{E1}+{\left(\varepsilon_{q\text{min}}^{C}\right)}^{E2}+{\left(\varepsilon_{q\text{min}}^{C}\right)}^{E3}+{\left(\varepsilon_{q\text{min}}^{C}\right)}^{E4} \end{array}$$

Once the type of reflector and the direction in which it is positioned are known, the approximate distance with respect to the sensor structure can be determined. The Frobenius norm in the transformed space between the feature vector ***τ****_e_* for the object to be classified, and every feature vector of the training samples of the class to which this reflector belongs, are calculated. For example, if the object was classified as a plane in the direction *q*, the TOF vector set used offline to generate the transformation matrix will have been $\left\{\tau_{q1}^{P},\tau_{q2}^{P},\tau_{q3}^{P},\ldots ,\tau_{qD}^{P}\right\}$. Therefore, it is only necessary to compute the Frobenius norm in the transformed space among the feature vector corresponding to the TOF vector ***τ****_e_*, and the feature vectors corresponding to the training samples, as is shown in:

(12) $$\varepsilon_{d}={\left\|L_{q}^{P^{T}}\Phi_{q}^{P}R_{q}^{P}\right\|}_{F}^{2}-{\left\|L_{q}^{P^{T}}\Phi_{d}R_{q}^{P}\right\|}_{F}^{2}$$

$\Phi_{d}=\tau_{\mathrm{qd}}^{P}-{\bar{\tau }}_{q}^{P}$, *d* = 1, 2,…, D.

The value corresponding to *d*, that provides the minimum *ε_d_*, will be the approximate reflector distance, in the direction *q*.

It has been proven empirically that the relationship among the distances of the reflectors to the sensor structure, and the Frobenius norm of their feature vectors in the transformed space, is approximately linear. In this way, considering the distance interval, where the reflector is, and the Frobenius norm in the transformed space, a correct estimation can be obtained by means of a linear interpolation of the distance at which the reflector is positioned.

### 3.3. Processing Time Using PCA and GPCA

To compare the processing time of the GPCA classification method with the PCA method, we analyze the number of multiplication operations required to classify the type of the reflector from the reconstruction error. To do this, we consider a generic sensor as proposed in Figure 2 with *r* rows and *c* columns. With TOF’s obtained a *r* × *c* matrix and a vector of dimension *r.c* are built for GPCA and PCA methods respectively. It’s also considered that a number *l* of eigenvectors are selected and that the sensor has *m* emitters.

In PCA, the reconstruction error is given by the following expression:

(13) $$\varepsilon =\left\|\Phi -\;\hat{\Phi }\right\|=\left\|\Phi -\;{\mathrm{LL}}^{T}\Phi \right\|$$

where L is the transformation matrix and **Φ** is the measurements column vector with mean zero. The dimensions of the vector **Φ** and matrix **L** are *r·c·m* (**Φ** ∈ ℜ^(^*^r.c.m^*^)^) and *(r·c·m)* × *l* (**L** ∈ ℜ^(^*^r.c.m^*^)×^*^l^*) respectively. We can obtain the number of multiplication operations required broking expression (13) down in different terms (14) and (15):

(14) $$L^{T}\Phi =E$$

(15) LE=F

Therefore, taking the dimensions of vector **Φ** and matrix **L** into account, to calculate the terms **E** = **L***^T^***Φ** and **F** = **LE** *l.(r.c.m)* multiplications are needed. To obtain the Euclidean distance, *r.c.m* multiplications are needed. So, the total number of multiplications required to classify the type of the reflector using PCA is:

(16) n° multiplications  (PCA)= l·(r·c·m)+l·(r⋅c·m)+(r·c·m)

In GPCA the reconstruction error is given by:

(17) $$\varepsilon ={\left\|\Phi \right\|}_{F}^{2}-{\left\|L^{T}\Phi R\right\|}_{F}^{2}$$

where **L** and **R** are transformation matrices and **Φ** is the measurements matrix with mean zero. In this case, the dimensions of matrices **Φ**, **L** and **R** are *r* × *c* (**Φ** ∈ ℜ^*r*×*c*^), *r* × *l*_1_ (L ∈ ℜ*^r^* ^×^ *^l^*^_1_^) and *c* × *l*_2_ (R ∈ ℜ*^r^* ^×^ *^l^*^_2_^ respectively. Following the same method that in PCA, we obtain the number of multiplications broking expression (17) down in terms:

(18) $$H=\Phi R\;\;\;\;\overset{n{}^{\circ}\;\mathrm{multiplications}}{\to }\;\;(r\cdot c\cdot l_{2}).m$$

(19) $$G=L^{T}H\;\;\;\;\overset{n{}^{\circ}\;\mathrm{multiplications}}{\to }\;\;\;(l_{1}\cdot r\cdot l_{2}).m$$

(20) $${\left\|G\right\|}_{F}^{2}\;\;\;\;\overset{n{}^{\circ}\;\mathrm{multiplications}}{\to }\;\;\;(l_{1}\cdot l_{2})\cdot m$$

(21) $${\left\|\Phi \right\|}_{F}^{2}\;\;\;\;\overset{n{}^{\circ}\;\mathrm{multiplications}}{\to }\;\;\;(r\cdot c)\cdot m$$

Then, the total number of multiplications required to classify the type of the reflector using GPCA is:

(22) $$n{}^{\circ}\;\text{multiplications}\;\;(\mathrm{GPCA})=\;\left[\left(r\cdot c\cdot l_{2})+(l_{1}.r.l_{2})+(l_{1}+l_{2})+(r\cdot c\right)\right].m$$

The number of multiplications for both methods particularized for *r* = 3, *c* = 3 and using different numbers of emitters (*m*) and eigenvalues (*l*, *l*_1_, *l*_2_) are shown in Table 1. One can see that the total number of multiplications using GPCA is slightly lower than using PCA. However, its main advantage is that because the classification is performed independently with the TOFS obtained for each emitter and added to the obtained values, it is possible to make a parallelization of the calculations when using a multiprocessor system. That is, for two emitters (*m* = 2) using a dual processor system, the processing time can be reduced by more than half using GPCA (160 using PCA and 68 using GPCA).

## 4. Simulation Results

A simulator has been used in order to carry out the simulations; this allows TOFs to be obtained in three-dimensional environments, based on the sensor model proposed by Barshan and Kuc [13] and using the rays technique [14]. The system employs a frequency of 50 Khz. To this frequency we can suppose a specular model. This simulator is the same as that used in [7] and it is validated with real measurements. The measures used to compare the two methods of classification, PCA and GPCA, have been obtained under the same conditions.

To evaluate and compare the GPCA classification method with the PCA method, we generate TOFs simulating the sensor structure of Figure 2. To obtain the transformation matrices, the reflectors have been located at distances from 50 to 350 cm, with 30-cm intervals. The azimuth and elevation angles were from −12° to +12°, with intervals of 2°.

In the simulations carried out, we analyze the percentage of successful classifications, using different values for the number of emitters (*m*) and different values of the number of used eigenvectors (*l* = *l*_1_ = *l*_2_) corresponding to the largest eigenvalues. In all the cases, the results are obtained adding to the TOFs a zero mean, independent and identically distributed (i.i.d.) Gaussian noise with typical deviation of 15 μs. The distance for plane, edge, and corner-type reflectors placed at distances from 50 to 350 cm, with intervals of 20 cm, and an azimuth angle of 7.5°. For each type of reflector at each distance 500 tests were conducted. We also performed simulations for different azimuth and elevation angles and the results are similar to the ones showed in this section.

**Figure 3.**
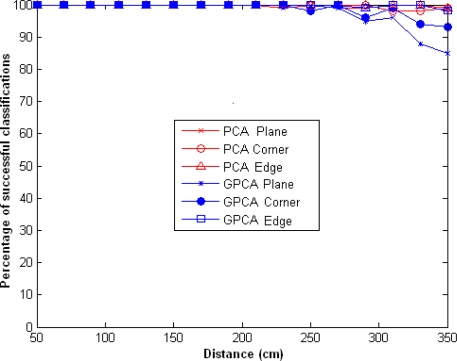
Percentage of successful classifications using GPCA and PCA, for *l* = *l*_1_ = *l*_2_ = 2 and *m* = 2.

In Figure 3 the classification percentage for *l* = 2 and *m* = 2, using PCA and GPCA methods, is shown. In this figure we can observe that the percentage of successful classifications using the PCA algorithm is greater than using the GPCA for distances greater than 200 cm. This is due to, the GPCA algorithm need a amount of input data greater than PCA for a appropriate classification. In both cases we obtained a 100% success rate for distances below 200 cm.

**Figure 4.**
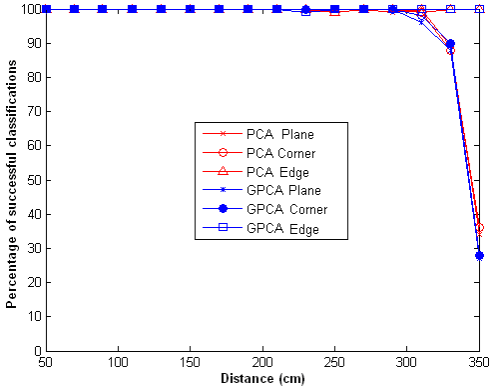
Percentage of successful classifications using GPCA and PCA, for *l* = *l*_1_ = *l*_2_ = 1 and *m* = 2.

If we maintain the same number of emitters and use only the eigenvector corresponding to the largest eigenvalue, the percentage of success is greater than 95% up to 300 cm and then decreases very sharply, as shown in Figure 4.

The simulations carry out with other values of noise and a single eigenvector have shown that, using PCA and GPCA the percentages successful classifications of corners and planes fall sharply for distances greater than 330 cm. This is due to the loss of dimensionality in the transformed space.

**Figure 5.**
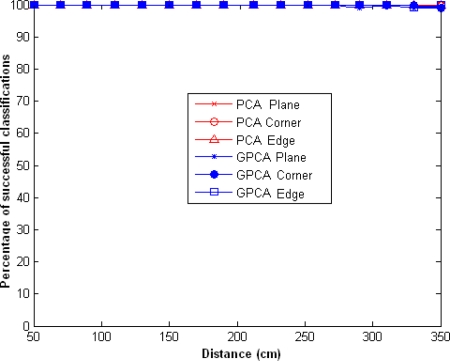
Percentage of successful classifications using GPCA and PCA, for *l* = *l*_1_ = *l*_2_ = 2 and *m* = 4.

If one wants to increase the percentage of hits in the classification, we can increase the number of emitters. In Figure 5 the results obtained for *m* = 4 are shown. In this figure we can see that the percentage of hits is over 98% up to 350 cm. In this case the processing time for PCA is approximately twice that with two transmitters. However, using GPCA we can get a time similar to that obtained for a single emitter, if we use parallel processing.

**Figure 6.**
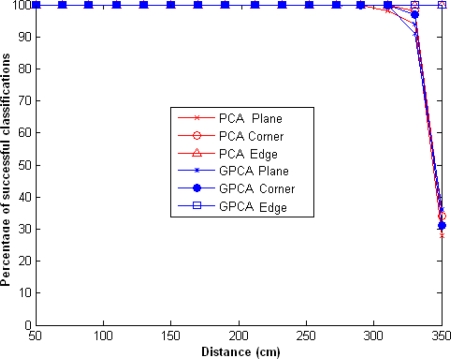
Percentage of successful classifications using GPCA and PCA, for *l* = *l*_1_ = *l*_2_ = 1 and *m* = 4.

In applications that do not require classifying objects at distances greater than 290 cm, you can use a single eigenvector to obtain 100% success. In Figure 6 the results obtained for *m* = 4 and *l* = 1 are shown. In this figure we can see that the percentage of success is 100% up to 290 cm and then decreases very sharply.

The GPCA approach better organizes the data in the sense of adjacent components in the matrix representation correspond to physically adjacent readings in the sensor array. This allows, with smaller transformation matrixes to achieve similar results to those obtained with PCA and the computational cost is lower.

The results obtained in estimating the distance are similar using PCA and GPCA methods. However, using GPCA has the advantage of a lower computational cost. The computational cost in the estimation of distance can be analyzed in the same way as is analyzed in the classification process.

Although all the results presented come from simulations done with non-correlated noise, tests with correlated noise have been carried out. It has been verified that the proposed algorithm is very robust against that kind of noise. In these tests, the classification is successful even when the correlated noise has higher standard deviations than the non-correlated noise.

## 5. Conclusions

In this paper, a classification algorithm based on the GPCA techniques have been proposed, with which three types of basic 3-D reflectors can be classified: planes, corners, and edges. The excellent behaviour of the GPCA proposed classification algorithm for a wide range of distances between reflectors and sensor systems has been demonstrated by means of simulations, under extreme noise conditions regarding measurements. Experiments show similar performance between GPCA and PCA, in terms of successful classification percentage. However, GPCA uses transformation matrices that are much smaller than PCA. This significantly reduces the space to store the transformation matrices and reduces the computational time in the classification procedure. Also, as the processing of the data is obtained for each emitter it is processed independently and processing time can be significantly reduced using parallel processing.

## Figures and Tables

**Table 1.** Number of multiplications for PCA and GPCA, using different numbers of emitters and eigenvalues.

**PCA**	**GPCA**	
***ε*** = ‖ **Φ** − **LL**^*T*^**Φ**‖	$\varepsilon ={\left\|\Phi \right\|}_{F}^{2}-{\left\|L^{T}\Phi R\right\|}_{F}^{2}$	Reconstruction error.
**Φ** ∈ ℜ ^(^*^r^*^·^*^c^*^·^*^m^*^)^	**Φ** ∈ ℜ*^r^*^×^*^c^*	Dimensions of matrices.
**L** ∈ ℜ ^(^*^r^*^·^*^c^*^·^*^m^*^)^*^×l^*	**L** ∈ ℜ*^r^*^×^*^l^*^_1_^	
	**R** ∈ ℜ*^c^*^×^*^l^*^_2_^	
*l* · (*r* · *c* · *m*) + (*r* · *c* · *m*)·*l* + (*r* · *c* · *m*)	(*r* · *c* · *l*_2_ + *l*_1_ · *r* · *l*_2_ + *l*_1_ · *l*_2_ + *r* · *c*) · *m*	Number of multiplication operations.
64 + 64 +32 = **160**	32 +16 + 4 + 16 = **68 × 2**	Number of multiplication operations for *l* = *l*_1_ = *l*_2_ = 2 and *m* = 2.
128 + 128 + 64 = **320**	32 +16 + 4 + 16 = **68 × 4**	Number of multiplication operations for *l* = *l*_1_ = *l*_2_ = 2 and *m* = 4.
32 + 32 + 32 = **96**	16 + 4 + 1 +16 = **37 × 2**	Number of multiplication operations for *l* = *l*_1_ = *l*_2_ = 1 and *m* = 2.
64 + 64 + 64 = **182**	16 + 4 + 1 +16 = **37 × 4**	Number of multiplication operations for *l* = *l*_1_ = *l*_2_ = 1 and *m* = 4.

## References

[1] Kleeman L., Kuc R. (1995). Mobile robot sonar for target localization and classification. Int. J. Robot. Res.

[2] Akbarally H., Kleeman L. A sonar sensor for accurate 3-D target localization and classification.

[3] Swets D.L., Weng J.J. (1996). Using discriminant eigen features for image retrieval. IEEE Trans. Pattern Anal. Mach. Intell.

[4] Malhi A., Gao R.X. (2004). PCA-based feature selection scheme for machine defect classification. IEEE Trans. Instrum. Meas.

[5] Yang J., Zhang D., Frangi A.F., Yang J. (2004). Two-dimensional PCA: A new approach to appearance-based face representation and recognition. IEEE Trans. Pattern Anal. Mach. Intell.

[6] Vázquez J.F., Lázaro J.L., Mazo M., Luna C.A. (2008). Sensor for object detection in railway environment. Sens. Lett.

[7] Jiménez J.A., Ureña J., Mazo M., Hernández A., Santiso E. (2005). Using PCA in time-of-flight vectors for reflectors recognition and 3-D localization. IEEE Trans. Robot.

[8] Ochoa A., Urena J., Hernandez A., Mazo M., Jimenez J.A., Perez M.C. (2009). Ultrasonic multitransducer system for classification and 3-D location of reflectors based on PCA. IEEE Trans. Instrum. Meas.

[9] Díaz V., Urena J., Mazo M., Jimenez J.A., García J.J., Bueno E., Hernandez A. Using complementary sequences for multimode Ultrasonic operation.

[10] De Marziani C., Ureña J., Hernández A., Mazo M., Álvarez F., García J.J., Donato P. (2007). Modular architecture for efficient generation and correlation of complementary set of sequences. IEEE Trans. Signal Process.

[11] Hernandez A., Urena J., García J.J., Mazo M., Dérutin J.P., Serot J. Ultrasonic sensor performance improvement using DSP-FPGA based architectures.

[12] Ye J., Janardan R., Li Q. GPCA: An efficient dimension reduction scheme for image compression and retrieval.

[13] Barshan B., Kuc R. (1990). Differentiating sonar reflections from corners and planes by employing an intelligent sensor. IEEE Trans. Pattern Anal. Mach. Intell.

[14] Watt A., Watt M. (1992). Advanced Animation and Rendering Techniques.

